# Supplementary material for: Positron Emission-Computed Tomography, Cryobiopsy versus Bronchoalveolar Lavage and Computed Tomography Findings for Interstitial Lung Disease: A Long-Term Follow-Up
Source: Medicina (Kaunas). 2023 Apr 18;59(4):787. doi: 10.3390/medicina59040787 (PMC10144839; doi:10.3390/medicina59040787)

Binomial logistic fit of a multiple regression for cryobiopsy vs survival, change of disease diagnosis and drug administration and respiratory functions after forward selection of the most important parameters.

Table S1. Whole Model Test.

Model	−LogLikelihood	DF	ChiSquare	Prob>ChiSq
Difference	14.388797	4	28.77759	<0.0001
Full	41.062978			
Reduced	55.451774			

In yellow: The change of diagnosis was statistical significant

AICc	92.9368
BIC	104.036
Observations	80

Table S2: Parameter Estimates

Term	Estimate	Std Error	ChiSquare	Prob>ChiSq
Intercept	−0.3718766	0.3607943	1.06	0.3027
change in Diagnosis[0]	−1.0207919	0.3566797	8.19	0.0042
survival[0]	−0.606382	0.2914361	4.33	0.0375
CHANGE IN DRUGS[0]	−0.5943222	0.3522594	2.85	0.0916
change in Diagnosis[0]*CHANGE IN DRUGS[0]	−1.0313838	0.3505591	8.66	0.0033

For log odds of 1/0, In yellow: The change of diagnosis was statistical significant; In red: The survival was positively associated with the change in diagnosis

Table S3: Odds Ratios

Odds Ratios for change in Diagnosis

Level1	Level2	Odds Ratio	Prob>Chisq	−95%	+95%
1	0	7.70	0.0042	1.90	31.18
0	1	0.13	0.0042	0.03	0.53

Odds Ratios for survival

Level1	/Level2	Odds Ratio	Prob>Chisq	−95%	+95%
1	0	3.36	0.0375	1.07	10.54
0	1	0.30	0.0375	0.09	0.93

Odds Ratios for CHANGE IN DRUGS

Level1	/Level2	Odds Ratio	Prob>Chisq	−95%	+95%
1	0	3.28	0.0916	0.83	13.06
0	1	0.30	0.0916	0.08	1.21

Normal approximations used for ratio confidence limits effects: change in Diagnosis survival CHANGE IN DRUGS. Tests and confidence intervals on odds ratios are Wald based. In red: The survival was positively associated with the change in diagnosis

Figure S1. Prediction profiler

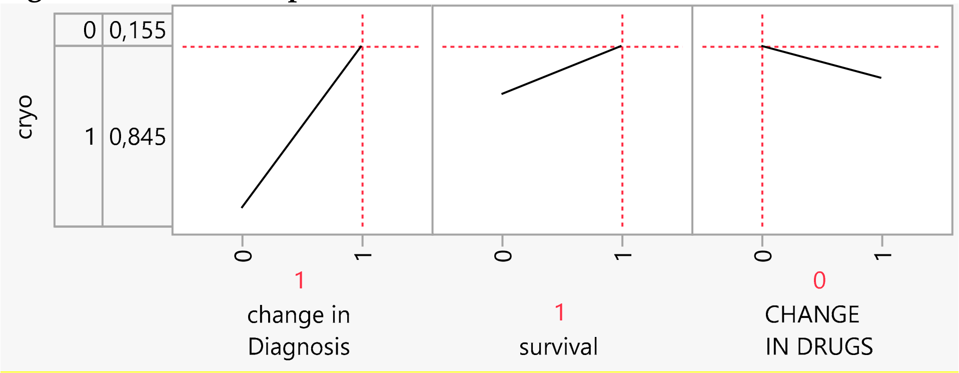

Figure S2. Interaction Profiles

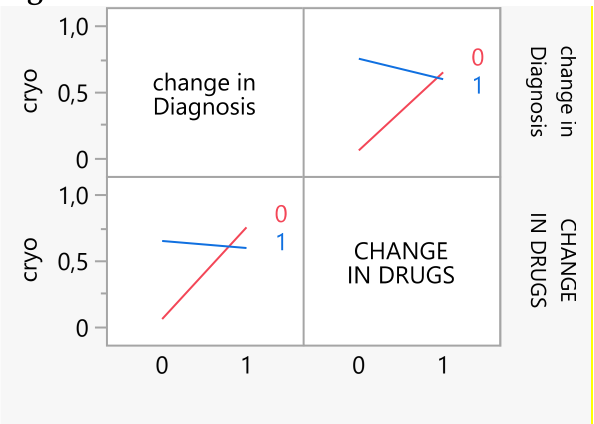

Figure S3. Studentized Deviance Residual by Predicted

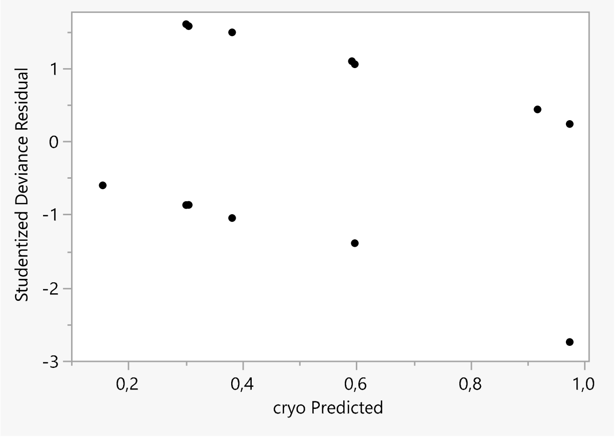

Supplement: Supplementary file 1 [file medicina-59-00787-s001.zip › medicina-2271526-supplementary.pdf]
